# From the *sxtA4* Gene to Saxitoxin Production: What Controls the Variability Among *Alexandrium minutum* and *Alexandrium pacificum* Strains?

**DOI:** 10.3389/fmicb.2021.613199

**Published:** 2021-02-24

**Authors:** Solène Geffroy, Marc-Marie Lechat, Mickael Le Gac, Georges-Augustin Rovillon, Dominique Marie, Estelle Bigeard, Florent Malo, Zouher Amzil, Laure Guillou, Amandine M. N. Caruana

**Affiliations:** ^1^IFREMER-Phycotoxins Laboratory, Nantes, France; ^2^IFREMER-DYNECO Pelagos, Plouzané, France; ^3^Sorbonne Université, CNRS, UMR 7144 Adaptation et Diversité en Milieu Marin, Station Biologique de Roscoff, Roscoff, France

**Keywords:** *Alexandrium*, saxitoxins, *sxtA4*, copy number variation, *sxtA*, expression, isoform

## Abstract

Paralytic shellfish poisoning (PSP) is a human foodborne syndrome caused by the consumption of shellfish that accumulate paralytic shellfish toxins (PSTs, saxitoxin group). In PST-producing dinoflagellates such as *Alexandrium* spp., toxin synthesis is encoded in the nuclear genome via a gene cluster (*sxt*). Toxin production is supposedly associated with the presence of a 4th domain in the *sxtA* gene (*sxtA4*), one of the core genes of the PST gene cluster. It is postulated that gene expression in dinoflagellates is partially constitutive, with both transcriptional and post-transcriptional processes potentially co-occurring. Therefore, gene structure and expression mode are two important features to explore in order to fully understand toxin production processes in dinoflagellates. In this study, we determined the intracellular toxin contents of twenty European *Alexandrium minutum* and *Alexandrium pacificum* strains that we compared with their genome size and *sxtA4* gene copy numbers. We observed a significant correlation between the *sxtA4* gene copy number and toxin content, as well as a moderate positive correlation between the *sxtA4* gene copy number and genome size. The 18 toxic strains had several *sxtA4* gene copies (9–187), whereas only one copy was found in the two observed non-toxin producing strains. Exploration of allelic frequencies and expression of *sxtA4* mRNA in 11 *A. minutum* strains showed both a differential expression and specific allelic forms in the non-toxic strains compared with the toxic ones. Also, the toxic strains exhibited a polymorphic *sxtA4* mRNA sequence between strains and between gene copies within strains. Finally, our study supported the hypothesis of a genetic determinism of toxin synthesis (i.e., the existence of several genetic isoforms of the *sxtA4* gene and their copy numbers), and was also consistent with the hypothesis that constitutive gene expression and moderation by transcriptional and post-transcriptional regulation mechanisms are the cause of the observed variability in the production of toxins by *A. minutum*.

## Introduction

The paralytic shellfish poisoning (PSP) syndrome is caused by the consumption of shellfish contaminated by toxins of the saxitoxin group (STX-group), also named paralytic shellfish toxins (PSTs) (Bricelj and Shumway, 1998). In mammals, these toxins act as blockers of voltage-dependent Na^+^ channels, inhibiting the transmission of neuronal signals (Cusick and Sayler, 2013). The first symptoms are tingling sensations in the lips, tongue and throat, and numbness of the face, which may progress in the most severe and acute cases of intoxication to paralysis, respiratory arrest or cardiovascular shock leading to death (Zhang et al., 2013; Hurley et al., 2014).

Among the 16 cyanobacterial and dinoflagellate genera PST producers (Yunes et al., 2009; Wiese et al., 2010; Borges et al., 2015; Savela et al., 2015; Caruana and Amzil, 2018), two species (*Alexandrium minutum* and *Alexandrium pacificum*) are known to form recurrent toxic blooms in shallow confined coastal waters along the French coasts (Giacobbe et al., 1996; Abdenadher et al., 2012; Anderson et al., 2012; Dhib et al., 2013; Guallar et al., 2017; Condie et al., 2019). STXs account for 57 molecules that include (Wiese et al., 2010) non-sulfated (NeoSTX, STX), mono-sulfated (GTX1/4, GTX2/3, B1, B2), di-sulfated (C1, C2, C4) or decarbamoylated (dcGTX2/3 and dcSTX) derivatives (Masselin et al., 2001; Touzet et al., 2007b; Masseret et al., 2009; Laabir et al., 2011, 2013; Hii et al., 2016). Highly toxic strains are correlated with the presence of low levels of sulfonate derivatives, i.e., STX, NeoSTX, and GTX1/4 (Botana et al., 2017). In addition, the toxin content per cell results from physiological (Anderson et al., 1990), environmental (such as abiotic and biotic factors (Hwang and Lu, 2000; Grzebyk et al., 2003; Laabir et al., 2011, 2013; Aguilera-Belmonte et al., 2013) and genetic factors (Kellmann et al., 2010; Stüken et al., 2015).

The candidate gene cluster for the PST biosynthetic pathway (*sxt*) was first identified in the 1980s from a cyanobacterial strain that produces PSTs, *Cylindrospermopsis raciborskii* T3 (Kellmann et al., 2008b). Thirty catalytic functions correspond to twenty-six proteins clustered within a single 35 kb genomic region (Kellmann et al., 2008a,b). Eight of these proteins, encoded by the *sxtA, sxtB, sxtD, sxtG, sxtS, sxtH/T, sxtU*, and *sxtI* genes, are directly involved in PST synthesis in cyanobacteria leading to the production of certain STX derivatives (dcSTX and/or STX) (Kellmann et al., 2008b; Moustafa et al., 2009; Hackett et al., 2013). Then *sxtI* allows the conversion from dcSTX to STX, while other proteins coded by the *sxtL, sxtN, sxtO, sxtR, sxtX, sxtW, sxtZ, sxtPER*, and *sxtACT* genes are involved in the synthesis of other PST analogs and in the transport of the toxin (Zhang et al., 2014, 2017; Wang et al., 2015; Verma et al., 2019). Although cyanobacteria and dinoflagellates are not closely related phylogenetically (prokaryotic vs. eukaryotic organisms), homologs of the cyanobacteria *sxt* gene cluster have been found in toxic dinoflagellates (Stüken et al., 2011; Hackett et al., 2013; Verma et al., 2019).

The *sxtA* gene is involved in the first step of toxin synthesis. However, whereas one unique and long mRNA version of this gene exists in cyanobacteria, two isoforms potentially in multiple copies are detected in the toxic dinoflagellates *A. fundyense, A. minutum, A. catenella*, and/or *A. pacificum.* These isoforms consist of one “short” isoform encoding three catalytic domains (*sxtA1-3*) (excluding a 4th domain that has no homolog in existing databases [amino acids 822–976, (Le Gac et al., 2016)] and a “long” one encoding four catalytic domains (*sxtA1-4*) (Stüken et al., 2011; Wang et al., 2020a). Based on the presence of the *sxtA4* domain, it is thought that the two isoforms are also present in *A. affine*, *A. australiense*, *A. ostenfeldii, A. tamarense* and the toxic dinoflagellates *G. catenatum* and *P. bahamense* (Stüken et al., 2011; Suikkanen et al., 2013; Murray et al., 2015; Wang et al., 2020a). The *sxtA4* domain is also found in the transcriptome of two non-toxic dinoflagellates, *Prorocentrum micans* and *Cochlodinium polykrikoides*, but is composed of a different sequence than those found in toxic *Alexandrium* species (Wang et al., 2020a,b).

Early on, it was suspected that the “long” mRNA isoform bearing the specific 4th *sxtA* domain (or *sxtA4*) was essential for toxin production, due to their under-expression in the non-toxic mutant *A. pacificum* ACHK-NT (Zhang et al., 2014; Verma et al., 2019). Suikkanen et al. (2013) additionally observed that the saxitoxin-producing strain of *A. ostenfeldii* contained *sxtA4* whereas the spirolide-producing strain of *A. ostenfeldii* did not. Furthermore, it has been shown that the presence of the *sxtA4* gene is essential for toxin production, and that the *sxtA4* copy numbers (CPNs) was strongly correlated with the toxin content in 15 strains of *A. minutum*. However, no correlation was found between the *sxtA4* CPNs and PSP content in three strains of *A. ostenfeldii* (Savela et al., 2016).

In field surveys, *sxtA4* qPCR assays are found to be effective for identifying PST-producing dinoflagellates from mixed samples (Murray et al., 2011, 2019; Gao et al., 2015; Penna et al., 2015; Ruvindy et al., 2018). Indeed, the presence of *sxtA4* seems to be a putative proxy to reveal the presence of toxic cells in blooms since there is a strong correlation between the detection of *sxtA4* and the presence of toxins in *A. ostenfeldii* and *A. pacificum* (Murray et al., 2011; Savela et al., 2016). However, it has been reported that several strains (one strain of *A. tamarense*, two strains of *A. australiense* and one mutant of *A. pacificum*) possess *sxtA4* without producing toxin (Murray et al., 2011, 2015; Stüken et al., 2011; Zhang et al., 2014), challenging the use of the *sxtA4* copy number as a proxy for the presence or level of toxin production.

The relationship between copy number of the *sxtA4* domain and toxin content remains unclear and, so far, the available results suggest that the determination of toxin production is complex, and involves constitutive gene expression associated with processes regulating the level of toxin production (Yang et al., 2010; Wiese et al., 2014; Zhang et al., 2017; Akbar et al., 2018). The use of several gene expression modes, including both transcriptional and post-transcriptional processes, are suspected in dinoflagellates (Hackett et al., 2013; McLean, 2013; Roy and Morse, 2013). Although this *sxtA4* domain is highly conserved between cyanobacteria (i.e., *Cylindrospermopsis raciborskii*, *Anabaena circinalis*, *Lyngbya wollei, Aphanizomenon flos-aquae*) and PST-producing dinoflagellates, multiple variants and highly variable gene copy numbers (CPN) of this 4th *sxtA* domain have been reported both between and within dinoflagellate species, raising questions regarding the universality of a gene regulation mode (Murray et al., 2011; Stüken et al., 2011, 2015; Savela et al., 2016; Mendoza-Flores et al., 2018; Verma et al., 2019).

In this study, we investigate how genetic determinism influences PST production in two *Alexandrium* species: (i) by describing the intra- and inter-specific variability of the *Alexandrium* strains in terms of either their genetic characteristics (gene CPN, expression, genetic variants) or their toxin production (content and profile) between twenty European strains of *A. pacificum* and *A. minutum*, (ii) by comparing the genome size and the 4th *sxtA* domain copy number to the toxin production and, (iii) based on the transcriptomic data generated for *A. minutum*, we additionally compare this toxin production to the *sxtA4* gene expression level and allelic frequencies in eleven strains. The goal of our study is to provide a better understanding of the toxin production in *Alexandrium*, in particular to clarify whether the presence of *sxtA4* is a criterion for PST production in *Alexandrium* and whether there is a relationship between *sxtA4* CPN and toxin content.

## Materials and Methods

### Strains and Culture Conditions

Monoclonal and xenic cultures of *A. minutum* and *A. pacificum* were established from European coast samples (Thau lagoon in French Mediterranean Sea, Atlantic Ocean and English Channel) (Table 1), and were identified by morphological criteria and by partial rRNA sequences ((ITS1, 5.8S, ITS2) for *A. minutum* (Dia et al., 2014; Le Gac et al., 2016) and the large subunit (LSU) for *A. pacificum* (Supplementary Table 1). Batch cultures were maintained in a autoclaved, filtered natural Mediterranean seawater (at a salinity of 38) and English Channel seawater (at a salinity of 35) enriched with L1 nutrients without the addition of silica (Guillard and Hargraves, 1993). They were exposed to a 12:12-h light: dark cycle and a photon flux density of 100 μmol photons m^–2^ s^–1^ (cool-white fluorescent light; Osram, Munich, Germany) at 18°C ± 1°C. The origins of the strains are listed in Table 1. Counts were performed using a particle counter (Beckman-Coulter Multisizer 3, Fullerton, CA, United States).

**TABLE 1:** List of the *A. minutum* and *A. pacificum* strains used, with their genome size, *sxtA4* CPN per genome and toxin composition content (means ± SD, *n* = 3).

Species	Strain	Location	Year	Genome size (pg)	*sxtA4* CPN genome^–^^1^	Total intracellular PSTs (fmol cell^–^^1^)	Toxin composition^*a*^
*A. minutum*	RCC3167	English Channel, Bay of Morlaix (France)	2010	27 ± 0.54	14 ± 4	1.7 ± 0.20	C1, C2, GTX2/3
	CH940x	English Channel, Cork harbor (Ireland)	2010	27 ± 0.65	13 ± 4	2.9 ± 0.3	GTX2/3
	RCC4871	North Atlantic Ocean, Bay of Brest (France)	2012	25 ± 0.44	10 ± 3	18 ± 5.2	C1, C2, GTX2/3 (dc-GTX2/3)
	RCC4872	North Atlantic Ocean, Bay of Brest (France)	2012	28 ± 0.61	16 ± 7	3.6 ± 0.60	C1, C2, GTX2/3 (dc-GTX2/3)
	RCC4890	North Atlantic Ocean, Bay of Brest (France)	2012	24 ± 0.72	20 ± 4	3.9 ± 1.2	C1, C2, GTX2/3 (dc-GTX2/3)
	RCC7037	North Atlantic Ocean, Bay of Brest (France)	2012	25 ± 0.55	19 ± 6	19 ± 3.5	C1, C2, GTX2/3 (dc-GTX2/3)
	RCC7038	North Atlantic Ocean, Bay of Brest (France)	2012	27 ± 0.48	14 ± 14	4.3 ± 2.7	C1, C2, GTX2/3 (dc-GTX2/3, NeoSTX)
	RCC7039	North Atlantic Ocean, Bay of Brest (France)	2011	25 ± 0.45	46 ± 1	8.7 ± 2.1	C1, C2, GTX2/3 (dc-GTX2/3)
	RCC3327	English Channel, Bay of Morlaix, (France)	2010	28 ± 0.50	9 ± 1	5.4 ± 0.6	C1, C2, GTX2/3 (dc-GTX2/3)
	RCC2645	North Atlantic Ocean, Bay of Concarneau (France)	2010	26 ± 0.47	1 ± 1	N.D.	–
	RCC2644	North Atlantic Ocean, Bay of Concarneau (France)	2010	27 ± 0.056	1	N.D.	–
*A. pacificum*	G6-7^*b*^	Mediterranean Sea, Tarragona (Spain)	2011	72 ± 1.3	61 ± 10	23 ± 4.6	C2, GTX4 (GTX5, C1, GTX3, NeoSTX)
	IFR-ACA-15	Mediterranean Sea, Thau lagoon (France)	2015	72 ± 3.0	187 ± 71	30 ± 8.3	C2, GTX4, GTX5 (C1, GTX3, NeoSTX)
	B9-1	Mediterranean Sea, Thau lagoon (France)	2007	74 ± 0.97	65 ± 21	5.5 ± 1.2	C2, GTX4 (GTX5, C1, GTX3)
	C11-4	Mediterranean Sea, Thau lagoon (France)	2007	75 ± 1.5	59 ± 21	16 ± 6.3	C2, GTX4 (GTX5, C1, dcGTX2)
	F5-4	Mediterranean Sea, Thau lagoon (France)	2007	76 ± 1.2	34 ± 17	1.7 ± 0.7	C2, GTX4 (GTX5, C1, GTX3, dcGTX2)
	G2-1	Mediterranean Sea, Thau lagoon (France)	2007	74 ± 1.7	43 ± 13	13 ± 3.9	C2, GTX4, GTX5 (C1, GTX3)
	H8-4	Mediterranean Sea, Thau lagoon (France)	2007	74 ± 1.3	148 ± 78	11 ± 3.7	C2, GTX4, GTX5 (C1, GTX2/3)
	C2-4	Mediterranean Sea, Thau lagoon (France)	2007	73 ± 0.75	88 ± 46	18 ± 3.5	C2, GTX4 (GTX5, C1, GTX2/3)
	IFR-ACA-17	Mediterranean Sea, Thau lagoon (France)	2017	74 ± 0.34	50 ± 36	4.3 ± 1.4	C2, GTX5 (GTX4, C1, GTX2)

*RCC = Roscoff Culture Collection (http://roscoff-culture-collection.org/). CPN, copy number per genome; SD, standard deviation; N.D., not detected;*

*^*a*^When toxins are given in parentheses, this indicates that trace amounts of the toxins were detected (<10%).*

*^*b*^All of the strains come from vegetative cells, except for strain G6-7, which is germinated from a cyst.*

### Batch Culture of *Alexandrium* Strains

Batch cultures in the late exponential growth phase were prepared in order to limit CPN variations over the growth phases (Stüken et al., 2011). Triplicate batch cultures of the *Alexandrium* strains were inoculated at an initial density of 5,000 cells mL^–1^ in 2 L Erlenmeyer flasks and incubated for 9–10 days for *A. minutum* and 6 days for *A. pacificum* to reach the end of the exponential growth phase (Supplementary Figure 1). At this time, samples were taken to measure the PSTs, CPN, and cell density.

### PST Analysis by Liquid Chromatography/Fluorescence Detection (LC/FLD)

Centrifugation was performed at 3,000 *g* for 8 min at 4°C in order to harvest 1.7 × 10^6^± 5.4 × 10^5^ *A. minutum* cells and 7.8 × 10^5^± 2.4 × 10^4^ *A. pacificum* cells. The pellets were extracted using the protocol described by Caruana et al. (2020). The toxin analyses were performed by LC/FLD based on the method given by Van De Riet et al. (2009) with slight modifications. Only the toxins with available standards were targeted. The mono-sulfated (GTX1/GTX4, GTX2/GTX3, B1, B2), decarbamoylated (dcGTX2/dcGTX3) and dcSTX, NeoSTX and STX toxins were separated using a reverse phase chromatography column (Zorbax Bonus RP, 3.5 μm, 4.6 × 150 mm) with a flow rate of 0.8 mL min^–1^. The di-sulfated toxins (C1, C2) were separated using a reverse phase chromatography column (BetaBasic 8, 5 μm, 4.6 × 250 mm) with a flow rate of 0.8 mL min^–1^. The pH and/or column temperature was/were optimized to separate dc-GTX2/dcGTX3, GTX5 (B1) and C1/C2. The toxin concentrations were quantified using a 6-point calibration curve of the reference standards from CNRC (Halifax, NS, Canada). Limits of detection (LOD) and quantification (LOQ) were provided in Supplementary Table 2 and strains showing no detectable traces of searched toxins were considered non-producing PST strains or non-toxic.

### gDNA Isolation and Quantification

A polycarbonate 12-μm pore-size filter (Nucleopore track-etched membrane, Whatman, Fisher Scientific, Pittsburgh, PA, United States) was used to aseptically filter 1.7 × 10^6^± 5.4 × 10^5^ *A. minutum* cells and 7.8 × 10^5^± 2.4 × 10^4^ *A. pacificum* cells. The cells were then rinsed three times with sterile L1 medium. Total gDNA was isolated from the 12 μm filter using the Nucleospin Plant II kit (Macherey Nagel, Hoerdt, France), according to the manufacturer’s instructions with minor modifications. These modifications involved resuspending each filter in 1 mL of PL1 buffer, using a vortex for 3 min and incubating for 60 min at 65°C with 25 μL RNase A. The silica membrane was washed three times with PW1 and PW2 and then dried 5 min at 65°C. Sample elution was performed twice after an incubation with 25 μL of PE buffer (5 min at 65°C). The gDNA was quantified using a NanoDrop^®^ spectrophotometer (A260/A280, NanoDrop^®^ Spectrophotometer ND-1000, Labtech International Ltd., Ringmer, United Kingdom). The quality of the gDNA was examined on a 1% agarose gel (25 min, 100V) stained with GelRed^TM^ (Biotium, United States).

### *sxtA4* Genomic Amplification and Cloning

Genomic amplifications were performed using 10 ng of isolated gDNA, 300 nM of primers (Supplementary Table 3) and 1× GoTaq^®^ Green Master Mix (Promega, Madison, WI, United States). After denaturation at 95°C for 2 min, amplification consisted of 35 cycles for 30 s at 95°C, 45 s at 67°C and 50 s at 72°C. A final step of elongation was done at 72°C for 5 min. The amplified DNA fragments were visualized on 0.8% agarose (25 min, 100V) stained with GelRed^TM^, cut out and cleaned using the PCR Clean-up Gel Extraction kit (Macherey Nagel, Hoerdt, France) according to the manufacturer’s instructions. After a quantification and quality control step, the PCR products were cloned into a pGEM-T Easy Vector (Promega, Madison, WI, United States) according to the manufacturer’s instructions and transfected into JM109 *E. coli* competent cells (Promega, Madison, WI, United States). Following blue/white screening, recombinant plasmid DNA was extracted using the Plasmid DNA purification kit (Macherey Nagel, Hoerdt, France) according to the manufacturer’s instructions and then sequenced (Eurofins MWG Operon, Ebersberg, Germany).

### Sequence Analysis and Primer Designs

The nucleotide and protein bioinformatic analyses of the recombinant plasmids were performed using Vector NTI 9.1.0 (Invitrogen, Invitrogen Corporation, Carlsbad, CA, United States). Similarity searches between the sequenced recombinant plasmids were done with BLASTN and BLASTX sequence alignments against the nucleotide and protein sequences in the available databases from GenBank^1^. Specific qPCR primers *sxtA4* were designed with Primer Express 3.0 (Applied Biosystems, Carlsbad, CA, United States) in the clone similarity sequence between *A. minutum* and *A. pacificum*, respectively (Supplementary Table 4). The sequence data from this article can be found in the GenBank/EMBL databanks and are listed in Supplementary Table 4 with their accession numbers.

### Quantitative PCR Experiments (qPCR)

All of the quantitative PCRs (qPCR) were performed on an MX3000p qPCR system (Agilent Technologies Inc., Santa Clara, CA, United States) with 96-well polypropylene plates (Agilent). All of the qPCR reactions were run with Brilliant III Ultra-Fast SYBR^®^ Green qPCR Master Mix (Agilent), using 1× SYBR^®^ Green qPCR Master Mix, 300 nM of each primer (Supplementary Table 3) (sxt072-MinuPaci/sxt0073) and a known quantity of gDNA (1 and 5 ng) or recombinant plasmid DNA. The amplification cycle consisted of a hot start, 3 min at 95°C, amplification, 40 cycles for 50 s at 95°C and 20 s at 60°C. The specificity of the PCR amplification was checked using a heat dissociation protocol, one cycle for 1 min at 95°C, 30 s at 60°C and 30 s at 95°C after the final cycle of PCR. For all of the qPCR experiments, standard curves were obtained using a 10-fold dilution series (ca. 0–1.10^7^ copies) of Spe I linearized recombinant plasmids (New England BioLabs, Ipswich, United Kingdom). The *sxtA4* inserts were generated from purified PCR products from the *A. minutum* and *A. pacificum* strains RCC2645, IFR-ACA-15 and B9-1 (Supplementary Table 4). The *sxtA4* purified PCR products containing ∼8.10^4^ and 8.10^5^ copies, were used as internal standards to verify qPCR efficiency. Three biological replicates were performed, each in four technical replicates. A negative control without DNA was included for each PCR mix. All of the assays showed a *R*^2^ > 0.98 with mean efficiency of 104% (min 102%, max 109%).

### *sxtA4* Copy Number Determination

The DNA found in the purified PCR products, samples and linearized recombinant plasmids was quantified with the Quant-iT^TM^PicoGreen^®^ dsDNA assay kit (Invitrogen, Carlsbad, CA, United States) according to the manufacturer’s instructions. For the *sxtA4* genes, the standard curves were established by relating the Log^10^ number of copies to the threshold cycle number (Ct). The linear regression equation was determined using the MX3000p qPCR system where *Iplate* is the intercept and S is the slope. According to the linear regression equation, the copy numbers in each reaction (*CPNr*) were calculated as described by Stüken et al. (2015), with *CPr* corresponding to the crossing point of the individual reaction. Subsequently, the copy numbers in each reaction per ng (*CPNng*) were estimated as follows: $C\,P\,N\,n\,g=\frac{C\,P\,N\,r}{D\,N\,A\,i\,n}$ where *DNA*in is the amount of input DNA in ng. The copy numbers per genome (*CPN*_*G*_) were calculated as follows: $C\,P\,N=C\,P\,N\,n\,g\times \frac{G\,s\,i\,z\,e}{1000}$where *Gsize* is the measured genome size of the strain in pg.

### *sxtA4* Analyses in the *A. minutum* Transcriptomic Database

The *A. minutum* strain-specific sequence and expression levels of *sxtA4* were obtained from a previously published RNAseq dataset (Le Gac et al., 2016). Each strain was collected at the end of the exponential phase and the mRNA was sequenced on an Illumina Hiseq PE 2× (100 bp). Normalized expression values are calculated as 2^rlog (coverage). The FreeBayes software (Garrison and Marth, 2012) was used to identify genetic variants. As *A. minutum* strains are haploid in our culture conditions, allelic variants within strains reflect the presence of genetically divergent gene copies within a given strain. Biallelic variants were considered when displaying a quality criterion >40 and when covered more than 20 times in each strain. For each variant site, the reference allelic frequency was calculated as the number of reads corresponding to the reference allele (i.e., as in the reference transcriptome) divided by the total number of reads covering the site. In the *A. minutum* reference transcriptome (https://doi.org/10.17882/45445), the *sxtA4* reference sequence was *comp112540_c0_seq1* (Supplementary Figure 3).

### Genome Size Measurements

Genome size measurements were estimated by flow cytometry using the modified protocol described by Marie et al. (2000), and samples were extracted with minor modifications detailed in Supplementary material (Supplementary Table 5). The samples were analyzed using a FACS Canto II system equipped with a 488 nm laser. The fluorescence emission of the IP was collected by the orange photomultiplier equipped with a 610 LP filter. Each sample was analyzed for 3 min at a rate of 55 μL min^–1^. Three analyses were performed for each sample. The average of the first peak of the culture was compared with that of the reference on the distribution resulting from the orange channel. The subsequent quantity of DNA was then calculated using the following formula: *DNAquantity*(*Gbp*) = (*Meansample*/*Meanreference*)×6.8, where 6.8 is the DNA content in picograms (pg) of diploid human cells. In order to convert the number of nucleotide pairs to picograms, the following formula given by Dolezel et al. (2003) is used: *DNAcontent*(*pg*) = *genomesize*(*Gbp*)/0.978.

### Data and Statistical Analyses

The statistical analyses were performed using RStudio 1.2.1578. All of the values were expressed as mean ± standard deviation (SD). The differences were considered significant when *p* < 0.05. A Student’s *t*-test was applied to identify statistically significant differences in the *sxtA4* normalized expression between the PST strain group and the non-PST strain group and to test the difference in genome size between the two species. The comparisons between the *sxtA4* CPNs and PSTs in both species were performed using the non-parametric Mann-Whitney *U* test (Mann and Whitney, 1947), as it was shown that the distribution was not normal. To test the various relationships between genome size, gene CPNs and total PSTs, Spearman’s rank correlation coefficient (S) was used (Chan, 2003; Akoglu, 2018).

## Results

### Saxitoxin-Group Profiles

**FIGURE 1:**
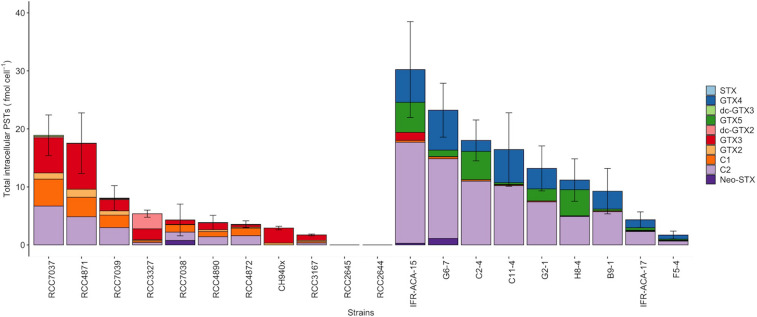
Intracellular STX-group content in the *Alexandrium minutum* **(left)** and *A. pacificum* **(right)** strains (including Neo-STX, dc-GTX2/3, GTX2/3, C1, C2, GTX1/4) (means ± SD, *n* = 3. Error bars represent standard deviations).

Among the 20 strains analyzed, two strains of *A. minutum* isolated from Concarneau Bay (France) were not toxic (RCC2644 and RCC2645), meaning that the targeted PST toxins for which there is a standard were not detected by our LC/FLD analysis (LOD values are detailed in Supplementary Table 1). Concerning toxic strains at the end of the exponential growth phase, the cellular toxin quota in the *A. pacificum* cells ranged from 1.7 ± 0.70 to 30 ± 8.3 fmol cell^–1^ (14 ± 3.7 fmol cell^–1^); this was comparable to the level found in the PST-producing *A. minutum* strains, which ranged from 1.7 ± 0.20 to 19 ± 3.5 fmol cell^–1^ (7.4 ± 1.8 fmol cell^–1^) (Table 1). Four main STX analogs were detected in the *A. minutum* strains (C1, C2, GTX2, GTX3), with the notable exception of strain CH940x which only contained GTX2 and GTX3 (Figure 1). Traces of Neo-SXT (RCC7038 only) and dcGTX2/3 (all of the *A. minutum* strains) were detected in all of the strains, with the exception of the RCC3167 and CH940x strains, which did not contain any *A. pacificum* strains contained C2 and GTX4, with the exception of strain C2-4 which contained C2 and GTX5 as dominant toxins and only traces of GTX4. Traces of dc-GTX2, GTX3, C1 and Neo-STX accounted for less than 10% of the total toxin content in the other strains.

### *sxtA4* Copy Number per Genome

**FIGURE 2:**
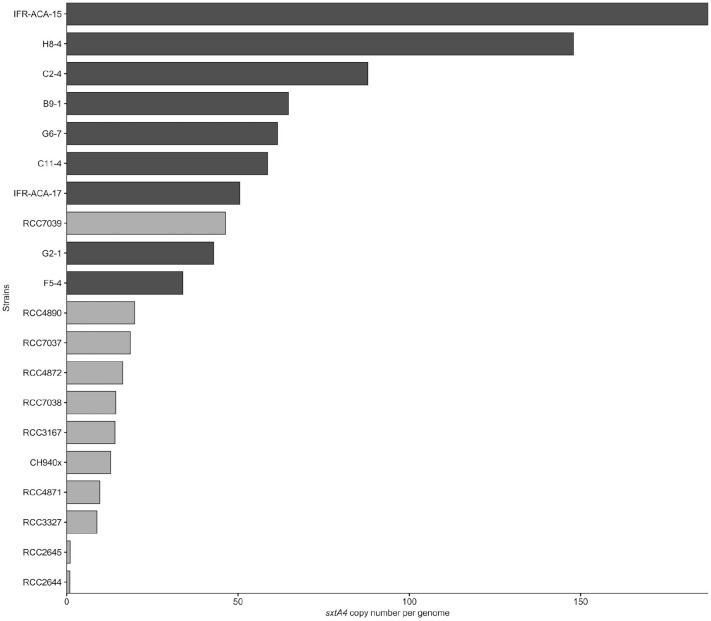
Average genomic *sxtA4* copy number in the *A. minutum* (light gray) and *A. pacificum* (dark gray) strains (*n* = 3).

**FIGURE 3:**
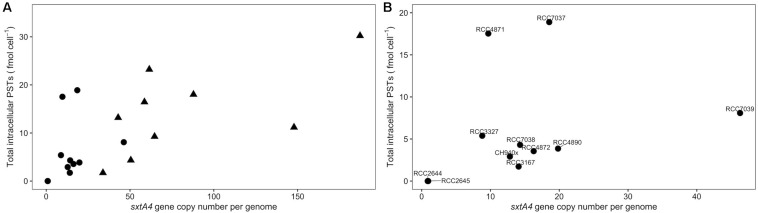
Scatterplots of the relationship between **(A)** the total intracellular toxins and the *sxtA4* copy number per genome in all of the strains (ρ = 0.58, *S* = 15085, *p* = 1.5 × 10^– 6^), and **(B)** specifically for *A. minutum* (ρ = 0.65, *S* = 3308, *p* = 0.009).

The *sxtA4* gene was amplified from all of the strains (toxin-producing and non-producing ones) but different copy numbers were detected. The *sxtA4* CPNs were significantly higher in *A. pacificum* compared with *A. minutum* (*U* test, *W* = 2, *p* < 0.001) (Figure 2). We counted between 9 and 46, and between 34 and 187 genomic copies of *sxtA4* in the toxic strains of *A. minutum* and *A. pacificum*, respectively (Table 1). In the two non-toxic *A. minutum* strains (RCC2644 and RCC2645), we counted a single *sxtA4* gene copy per genome. An overall positive relationship was statistically supported between the *sxtA4* CPN per genome and the total toxin content per cell (Spearman test, ρ = 0.58, *S* = 15085, *p* < 0.001) (Figure 3A). However, this correlation was more robust for *A. minutum* than for *A. pacificum* (Spearman test; *A. minutum*, ρ = 0.45, *S* = 3308, *p* < 0.01; *A. pacificum*, ρ = 0.47, *S* = 1736, *p* < 0.05). The RCC7037 and RCC4871 *A. minutum* strains contrasted with this general rule as they had high PST contents but a somewhat mid-range CPN value (Figure 3B). No correlations were detected between the *sxtA4* CPN and the year of isolation or geographical origin of the strains. For instance, the *sxtA4* CPN ranged from 34 ± 17 to 187 ± 71 in eight strains of *A. pacificum* isolated in the same year (2017) and from the same location (Thau Lagoon, Mediterranean Sea).

### Genome Size

**FIGURE 4:**
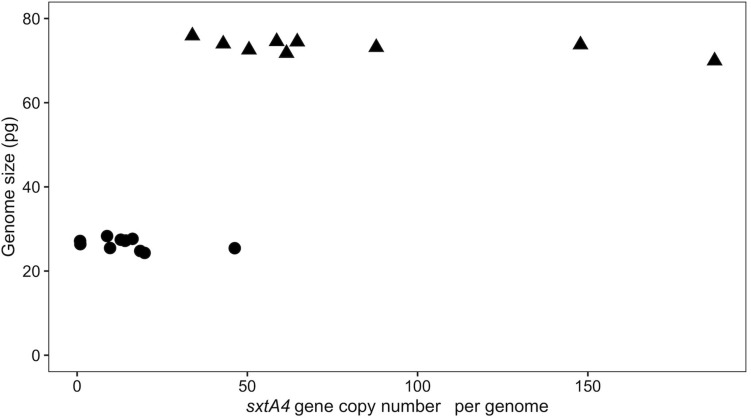
Scatterplots of the correlation between genome size and the *sxtA4* CPN (ρ = 0.58, *S* = 564, *p* = 0.009) for the *A. minutum* (circles) and *A. pacificum* (triangles) strains.

*Alexandrium pacificum* had a genome size that was three times larger than that of *A. minutum* (24 ± 0.70 and 76 ± 1.2 pg, respectively), with low intraspecific variation (*t*-test, *t* = 66.6, df = 14.5, *p* < 0.001) (Table 1). These values are in the range of those previously reported for other *Alexandrium* species, e.g., from 21.8 pg in *A. andersonii* to up to 204.5 pg in certain strains of *A. tamarense* (LaJeunesse et al., 2005; Figueroa et al., 2014; Casabianca et al., 2017). A positive correlation was found between the genome size and *sxtA4* CPNs when the 20 strains were considered (Spearman’s coefficient, ρ = 0.58, *S* = 15817, *p* < 0.001) (Figure 4). However, this correlation would no longer be supported if only a single species was considered due to a difference in the genomic size between *A. minutum* and *A. pacificum* (Supplementary Figure 2B).

### *A. minutum sxtA4* Gene Expression and Genetic Variation

**FIGURE 5:**
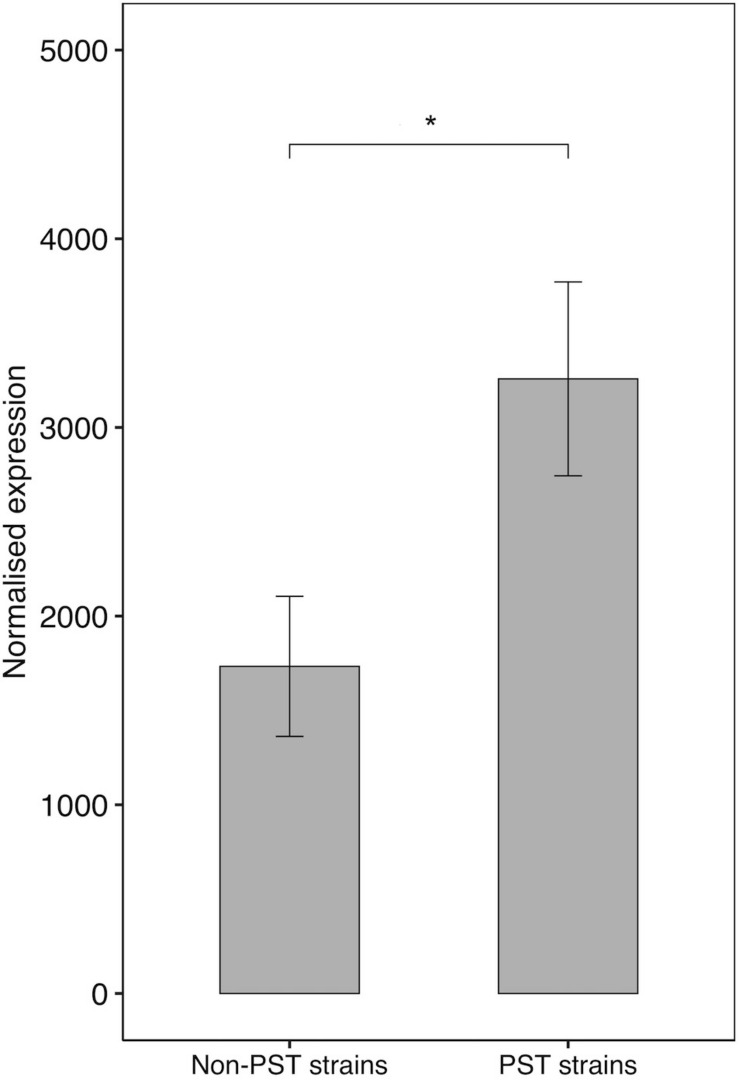
*SxtA4* normalized expression (from comp112540_c0_seq1) in the non-PST producing *A. minutum* strains (RCC2644 and RCC2645) and the PST-producing *A. minutum* strains (RCC3327, RCC7038, CH940x, RCC3167, RCC7037, RCC4871, RCC7039, RCC4872, and RCC4890). *T*-test, *p < 0.05. Error bars represent the standard deviations.

**FIGURE 6:**
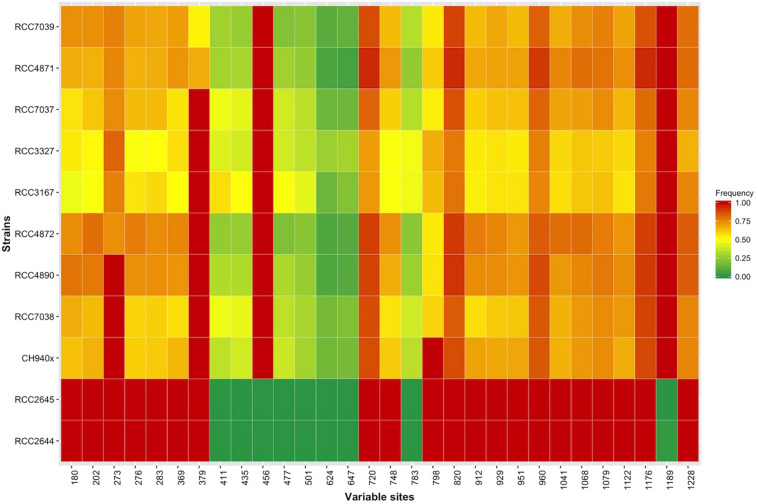
Heatmap showing the allelic frequencies at the 30 genetically variable sites in the *sxtA4* sequence (comp112540_c0_seq1) for each *A. minutum* strain.

**FIGURE 7:**
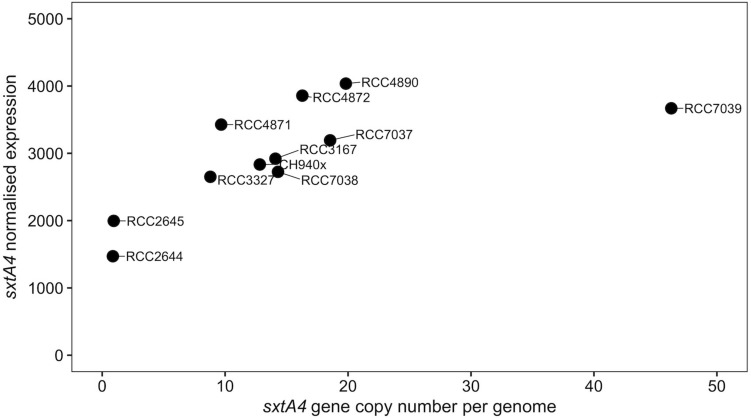
Scatterplot of the correlation between the *sxtA4* copy number per genome and the *sxtA4* normalized expression in the *A. minutum* strains (ρ = 0.82, *S* = 38, *p* = 0.003).

The *sxtA4* gene expression level was examined in two non-toxic strains and nine toxic strains. The *sxtA4* expression in the non-toxic strains was significantly lower than in the toxic strains (1733 ± 371.4 vs. 3257 ± 513.9, respectively, *t*-test, *p* < 0.05) (Figure 5). In all of the eleven *A. minutum* strains investigated, a total of 30 variable sites were detected on comparison with the reference *sxtA4* sequence (Figure 6). The nine toxic strains exhibited polymorphic *sxtA4* copies with some conserved parts and variable sites that differed between the strains. The toxic strains had between two to five invariable sites (i.e., reference allelic frequency of 0 or 1). For instance, the CH940x strain, which bore five invariable sites, only produced two toxins (GTX2 and GTX3). Whereas the nine PST-producing strains were polymorphic, the two non-producing strains had a monomorphic *sxtA4* gene, which was identical for the two strains. We also noted that two genetic positions (positions 456 and 1189) in the *sxtA4* mRNA sequence tended to display fixed differences between the PST-producing and non-producing strains. In these *A. minutum* strains, a strong significant positive correlation was found between the *sxtA4* CPN per genome and the normalized *sxtA4* expression (ρ = 0.73, *S* = 58.63, *p* = 0.01) (Figure 7).

## Discussion

### Interspecific Variability in Toxin Phenotype

*Alexandrium minutum* and *A. pacificum* are two well established species that differ in their toxin profiles (Balech, 1995; Laabir et al., 2007). The dominant saxitoxin isoforms found here in the French *A. pacificum* strains (GTX4, C2, and/or GTX5) are in line with the toxin profiles reported for the various strains found in the North Mediterranean Sea (Laabir et al., 2013; Lugliè et al., 2017), and differ from populations of the South Mediterranean Sea (dominated by GTX6 or C1 or GTX1, Hadjadji et al., 2020), Australian (yielded mostly GTX5 and GTX6, GTX1/4 and C1/2, Ajani et al., 2017) and Asian populations (Laabir et al., 2013). Similarly, the species *A*. *minutum* also presented diverse toxin profiles that were categorized into five clusters by Lewis et al. (2018). All of the strains originating from northwestern France (Morlaix and Brest) corresponded well with cluster 4, which gathered *A. minutum* species from Northern Europe characterized by a significant amount of C1/2 and GTX2/GTX3 toxins (Grzebyk et al., 2003; Nascimento et al., 2005; Touzet et al., 2007a,b, 2008). The Cork strain likely belonged to cluster 2, which included strains producing high levels of GTX2/3 from Northern Europe, especially from Ireland. Two strains from Concarneau Bay did not contain any targeted toxins (RCC2644 and RCC2645). It is uncommon to find non-toxic strains within primarily toxic species, although this had already been reported in *A. australiens*e, *A. pacificum*, *A. ostenfeldii*, *A. minutum*, and *A. affine* (Nguyen-Ngoc, 2004; Touzet et al., 2007a; Brown et al., 2010; Yang et al., 2010; Murray et al., 2012; Suikkanen et al., 2013; Zou et al., 2014). However, RCC2644 and RCC2645 were still highlighted as being independent genetic entities from other *A. minutum* strains originating from Cork harbor or the coasts in northwestern France (Le Gac et al., 2016). One possible scenario explaining this situation would be an ancestral divergence via complete isolation followed by a secondary contact involving gene flow (Le Gac et al., 2016). The presence of a ventral pore on the right side of the 10 plate was pointed out as a useful diagnostic phenotypic characteristic that can be used to identify this subgroup of *A. minutum* strains originating from Concarneau (southern Brittany, France). Our study highlights an absence of known toxin production as another likely distinctive feature of this subgroup of *A. minutum* strains, which could constitute a 6th cluster in the *A. minutum* populations. Both *A. minutum* and *A. pacificum* exhibited various toxin profiles among the studied populations and across their biogeographical distributions, potentially calling their descriptions as unique species into question.

### Variability in *sxtA4* Copy Numbers and Genome Size

Considering the *A. minutum* and *A. pacificum* strains all together, our results suggest a continuum between genome size, gene copy number and toxin content. Hence, in the larger cells of *A. pacificum*, the larger genome contained more elevated *sxtA4* CPNs and a higher toxin content than in the smaller cells of *A. minutum*. A significant difference in the number of *sxtA4* gene copies was observed here between the species, with a four-fold lower *sxtA4* CPNs in the toxin-producing strains of *A. minutum* (9–46) than *A. pacificum* strains (34–187). These observations are congruent with previous publications reporting variable *sxtA4* CPNs depending on the strains and species (Table 2). The *sxtA4* CPNs for *A. minutum* reported in this study (9–46 genomic copies) were higher than those reported by Stüken et al. (2015), (1.5 to 10.8 genomic *sxtA4* CPN). By contrast, similar values were reported for *A. pacificum* in this study (34–187 *sxtA4* CPN) and for the Australian strains (100–280 *sxtA4* CPN) (Murray et al., 2011, 2015; Stüken et al., 2011). In *Gymnodinium catenatum*, which produces PST concentrations up to 122 fmol cell^–1^ (Montoya et al., 2006), 74 to 143 copies cell^–1^ were reported in one strain for different growth phases (Mendoza-Flores et al., 2018). No correlation was detected here, between the *sxtA4* CPN and the year of isolation or geographical origin of the strains (for instance, 34–187 *sxtA4* CPNs for eight *A. pacificum* strains isolated in Thau Lagoon in 2017).

**TABLE 2:** *sxtA4* CPN per genome and toxin content from PST-producing species reported in the literature.

Alexandrium species	Strain name	Geographic origin	Culture or environmental sample	Culture status	Genomic *sxtA4* copies	Cell toxin quotas (fmol cell^–1^)	References
*Alexandrium minutum*	RCC3167, CH940x, RCC4871, RCC4872, RCC4890, RCC7037, RCC7038, RCC7039, RCC3327, RCC2645, RCC2644	Brittany (France), Cork (Ireland)	Cultured	End of exponential growth phase	1–46	0–19	This study
	CCMP113, VGO722, AMP13, AL1OC, VGO942, AL4V, Min3, VGO577, AMP4, VGO874, RCC3227, RCC3337, VGO650, VGO651, VGO663	Mediterranean Sea (Spain, Italy, Tunisia), Brittany (France)	Cultured	End of exponential growth phase	1.5–10.8	1.850–17.77	Stüken et al., 2015
*Alexandrium pacificum*	IFR-ACA-15, IFR-ACA-17, B9-1, C11-4, F5-4, G2-1, H8-4; C2-4	Mediterranean Sea, Thau lagoon (France)	Cultured	End of exponential growth phase	34–187	1.7–30	This study
	G6-7	Mediterranean Sea, Tarragona (Spain)	Cultured	End of exponential growth phase	61	23	This study
	ACSH02	Sydney Harbor (Australia)	Cultured	Early exponential phase, late and stationary phase	100–240	-	Stüken et al., 2011
	ACSH02^1^, ACCC01^2^, ACTRA02^3^	Sydney Harbor^1^, Cowan Creek^2^; Tasmania^3^, (Australia)	Cultured	End of exponential growth phase or early stationary phase	178–280	3.1-6.6 pg cell^–1^	Murray et al., 2011
	-	Brisbane Water, Georges River and Wagonga Inlet (Australia)	Environmental sample	-	226–376 *sxtA4* cell^–1^	-	Murray et al., 2011
*Alexandrium ostenfeldii*	AOF 0924, AOF 0935, AOKAL 0902, AOPL 0914	Baltic Sea	Cultured	-	1–11	-	Savela et al., 2016
	-	Föglö, Aland (Baltic Sea)	Environmental sample	-	2.5 x 10^4^ - 3.0 x10^9^ cells L^–1^	4–41 pg cell^–1^	Savela et al., 2016
*Gymnodinium catenatum*	LC-62	Lázaro Cardenas (Mexican Pacific)	Cultured	Different growth phase	74–143	-	Mendoza-Flores et al., 2018

*^*a*^except for G6-7 from Tarragona (Spain) in the Mediterranean Sea.*

The numerous *sxtA4* copies found in these two species propose that toxin genes were conserved throughout the genomic replication occurring during the evolution of dinoflagellates, in particular *A. minutum* and *A. pacificum* species (Lin, 2011). The multiple gene copies are not restricted to toxins in dinoflagellates, and the number of gene copies may be elevated and highly variable both within and between species. This was the case for gene encoding rRNA (2,489,800 ± 550,967 *A. pacificum* CNR-ACATS3 and 1345 ± 780 in *A. tayl*ori CBA-1), luciferase (44–160 copies), the peridinin-chlorophyll *a*-binding protein (36–5000), protein kinase (30), actin (>113 gene copies), proliferating cell nuclear antigens (191.87 ± 32.13) and even form II RuBisCO (117 ± 40, 148 ± 16), which were detected in a wide range of copy-number interspecies of the genera *Alexandrium*, *Protoceratium, Prorocentrum*, and *Lingulodinium* (Le et al., 1997; Salois and Morse, 1997; Zhang and Lin, 2003; Liu and Hastings, 2005; Galluzzi et al., 2010; Shi et al., 2013; Hou et al., 2019). Further, the comparison of *sxtA4* gene copy number with the copy number of other genes in *A. minutum* and *A. pacificum* would give a better comprehension on the conservation and evolution of toxin genes in the whole genome.

The results of our study support the hypothesis of a direct link between *sxtA4* CPN and toxin content in the species *A. minutum* and *A. pacificum* as proposed by some authors on *A. minutum* (Stüken et al., 2015). This indicated that the *sxtA4* CPN partially determined the amount of toxins produced in these species. However, with the exception of three strains (*A. minutum* RCC7037, RCC7039, and *A. pacificum* H8-4) that showed a weak correlation between the *sxtA4* CPN and the toxin content. Moreover the correlation does not exist for some other *Alexandrium* species such as *A. ostenfeldii* strains, which contain a high level of intracellular toxin (4 to 41 pg cell^–1^) but only a few copies of the *sxtA4* gene (∼ 6 copies per genome), indicating no direct relationships between PST production and cellular *sxtA4* copies in this species (Savela et al., 2016). The accumulation of non-functional *sxtA4* copies and/or additional regulation processes at the transcriptional and post-transcriptional levels might also explain a divergence between the number of *sxtA4* copies and the quantity of toxins produced in *A. minutum* and *A. pacificum*.

The genome size appeared to have a lower intra-specific variability (24–27 and 72–76 pg), compared with what was observed for the toxin content (1.7–19 and 1.7–30 fmol cell^–1^) and *sxtA4* CPN (9–46 and 34–187) within the *A. minutum* vs. *A. pacificum* strains, respectively, indicating other evolutionary constraints for genome size. The genome size of dinoflagellates is particularly large compared with other free-living Myzozoa (chromerids, up to 193 Mb; LaJeunesse et al., 2005; Stüken et al., 2015; Woo et al., 2015), but is rather homogeneous within strains of a single species in our data. This observation can be explained by a process accumulating genomic material while maintaining a rather constant genome size. The interspecific variations in genome sizes, DNA content and the copy number of *sxtA4* genes between *A. minutum* and *A. pacificum* have been potentially explained by genome duplications and polyploidization (Loper et al., 1980; Stüken et al., 2015). However, polyploidy was refuted based on genome analysis of four *Symbiodinium* species (Lin et al., 2015; Liu et al., 2018), and would considerably scatter the genome size values within a given species. Other evolutionary processes may explain a constrained but large genome size and intraspecific variabilities in terms of CPNs in dinoflagellates such as segmental duplication by unequal crossover, may be favored by the permanently condensed dinoflagellate chromosomes, and/or retroposition mechanisms via the reverse transcription of mRNAs and their re-integration within the genome (Smith, 1976; Jaeckisch et al., 2011; Hou et al., 2019).

The presence of the 4th domain of the *sxtA* gene in the genome of *Alexandrium* spp. seems to be a good proxy for the capacity of a given strain to produce saxitoxins, with few exceptions reported so far (i.e., *A. minutum* RCC2644, RCC2645; *A. australiense* ATCJ33, ATEB01; *A. tamarense* CCMP1771; and the mutant *A. pacificum* ACHK-NT) (Murray et al., 2011; Stüken et al., 2011; Zhang et al., 2014). In the European *A. minutum* and *A. pacificum* strains, the genomic copy number of *sxtA4* also appears to be a good indicator of the toxin production level, tough it remains an ambiguous marker for other species. Moreover, given that there is a potential relationship between the genome size, the gene copy number and toxin quotas in dinoflagellates, the compilation of a larger dataset on PST-producing strains of dinoflagellates is encouraged so as to further assess this observation.

### *sxtA4* Expression and Isoforms in *A. minutum*

The nine toxin-producing strains with several (>9) *sxtA4* copies were genetically polymorphic, with several divergent *sxtA4* copies co-occurring within a given strain. The observed variability in the toxin phenotypes (in terms of content) may result from the polymorphism of the *sxtA4* copies that exists among the strains. Also, the polymorphism seems to be a crucial feature for toxin production, since a higher number of fixed sites was associated with lower toxin diversity (as found in the strain CH940x).

We reported two novel strains of non-toxic *A. minutum* (RCC2644 and RCC2645) bearing a unique *sxtA4* copy. Two mutations, located at two distinct positions (456 and 1186), could be used to distinguish between toxin- and non-toxin producers. These mutations did not disrupt the transcription into mRNA since the *sxtA4* gene was expressed in the two non-toxic strains, though in fewer copies. These results demonstrate that the absence of targeted toxin production in strains RCC2644 and RC2645 was not due to the absence of domain 4 of the *sxtA* gene but to the presence of an atypical isoform of *sxtA4* in a small number of copies.

Otherwise, a positive relationship was observed between the *sxtA4* CPN and the mRNA expression levels in this study and, for the first time, a direct relationship between *sxtA4* mRNA expression and the toxin content was observed for all *A. minutum* strains. Again, this indicates that the *sxtA4* CPN in the genome may contribute in determining the amount of toxins produced by *A. minutum* species, an observation that supports constitutive gene expression.

Moreover, cases of discrepancies between the CPN and toxin amounts suggested that transcription processes play also a major role in the regulation of STX-group synthesis, as already suggested by Bachvaroff and Place (2008). For instance, here, four-fold higher *sxtA4* CPNs in *A. pacificum* was associated with only 1.5-fold higher toxin content than in *A. minutum*. Also as detailed above, our results showed a weak correlation for three strains (*A. minutum* RCC7037 and RCC7039, *A. pacificum* H8-4) and similarly, another study reported no correlation for *A. ostenfeldii*, in which a high level of intracellular toxin content (4 to 41 pg cell^–1^) was associated with a low *sxtA4* CPN (∼6 copies per genome) (Savela et al., 2016).

Furthermore, although Zhang et al. (2014) reported the down-regulation of sxtA4 mRNA associated with the absence of toxin production in the mutant ACHK-NT, most studies were unable to identify a correlation between the expression level and the amount of toxin, suggesting the concomitant participation of other expression-modifying mechanisms such as translational and post-translational mechanisms. For instance, no significant variations in the expression level of toxin-related genes were observed in *A. pacificum* strain ACHK-T (*sxtB, sxtD, sxtF/M, sxtG, sxtH/T, sxtI, sxtO, sxtP, sxtU, sxtW, sxtX, sxtZ, sxtPER*) (Zhang et al., 2017) or in *A. pacificum* ACCC01 (*sxtA4*) during the toxin-producing phases (Wiese et al., 2014). Similarly, no significant variations in the expression level were found in the two strains of toxin-producing cyanobacteria, *Aphanizomenon gracile* (*sxtA, sxtM, sxtPer*) and *Raphidiopsis brookii* (*sxtU, sxtI)* (Vico et al., 2016; Cirés et al., 2017). Moreover, no correlation was found between the toxins and the mRNA amounts for *sxtA1* and *sxtG* in *A. minutum* (Perini et al., 2014).

In dinoflagellates, only 5 to 30% of the genes seem to be regulated at the transcription level; the remaining genes are supposedly regulated post-transcriptionally (Lidie et al., 2005; Erdner and Anderson, 2006). Involved processes could be mRNA editing (Scott, 1995; Lin et al., 2002, 2007; Zauner et al., 2004; Dang and Green, 2009; Mungpakdee et al., 2014), the participation of small interfering RNA (RNAsi), recently discovered in dinoflagellates (Zhang and Lin, 2019), which might affect *sxtA4* expression. RNAsi hybridizes to mRNA, which leads to its degradation and alters gene expression, either by suppressing gene expression or through a gene regulatory network.

In addition to transcriptional mechanisms, translational or post-translational mechanisms (phosphorylation, methylation, and glycosylation, protein cleavage) could modify the transport of mRNAs, the translation of mRNAs into proteins, or the protein molecule itself (Akbar et al., 2020). For instance, in dinoflagellates several translational and post-translational mechanisms have been found to regulate proteins involved in bioluminescence (Mittag et al., 1994, 1998), and NADP-ICDH in circadian rhythms (Akimoto et al., 2005) in *Lingulodinium polyedrum* and in the cell cycle in *Karenia brevis* (Brunelle and Van Dolah, 2011).

Hence, several transcriptional and post-transcriptional processes may co-exist in dinoflagellates and explain why the expression of a single *sxtA4* gene copy in *A. minutum*, even at a low level, does not lead to the production of saxitoxin in our two non-toxic strains. Nevertheless, in our French toxic strains of *A. minutum*, all of the potential regulation mechanisms were minimized in favor of constitutive gene expression for toxin production.

## Conclusion

This study provides new data on the involvement of the *sxtA4* gene in toxin production, as well as a deeper understanding of STX-group synthesis in dinoflagellates. Correlations were observed by comparing data from two independent experiments at the transcriptomic and genomic levels on the same strains of *A. minutum* (analyses of *sxtA4* transcripts vs. analyses of the number of copies of the *sxtA4* gene and the toxin content). In particular, we observed that the number of *sxtA4* gene copies, the presence of genetic isoforms, and the level of the *sxtA4* mRNAs expression profiles determined the toxin content of a strain. However, it remains to distinguish between the presence and the functionality of *sxtA4* copies to explain potential discrepancies between high gene copy number and toxin content. Moreover, non-toxin producers are genetically distinct from other *A. minutum* strains, with a unique *sxtA4* gene allele (isoform) detected in a single copy. Expression of this gene still occurs, although to a lesser extent compared with other toxin-producing strains. These findings are promising and need to be further screened on a larger panel of non-toxic strains to determine the products of this *sxtA4* isoform. Moreover, the polymorphism observed in the *sxtA4* copies, both among strains and within one strain, is likely to be related to the interspecies variability in the toxin content. Further investigations of the *Alexandrium sxt* genes are warranted to understand how *sxt* genes work together to produce diverse toxin profiles in *Alexandrium*.

Nonetheless, the inconsistencies encountered in some strains in terms of the CPN, expression rate and toxin contents highlight the genomic complexity of dinoflagellates, which likely regulates their gene expression at the genomic, transcriptional and translational levels. Understanding the underlying mechanisms will hopefully provide a better explanation of the observed intra- and interspecies phenotypic diversity. In the future, the expression level of PST-producing dinoflagellates should be compared with proteomic and metabolic analyses. Whereas at the environmental level, recent qPCR tests targeting the *sxtA4* gene have been proven to be sensitive and to produce efficient results in terms of estimating the abundance of toxic *Alexandrium* cells, our results highlight the relevance of developing probes that target the various *sxtA4* isoforms in order to identify toxic and non-toxic individuals within the same population.

## Data Availability Statement

The datasets presented in this study can be found in online GenBank/EMBL repository. The accession number(s) can be found in the in theSupplementary Material (Supplementary Table 4).

## Author Contributions

SG, M-ML, AC, LG, and ZA conceived, designed, and reviewed the study. SG carried out all experimental work, acquired, analyzed, interpreted data, and drafted the manuscript. M-ML and EB participated in the experimental design of the qPCR analysis. ML performed the transcriptomic analysis. G-AR conceived and validated the toxins analysis method. DM analyzed the genome size. FM isolated and maintained the strains. All authors contributed to the writing and editing article and approved the submitted version.

## Conflict of Interest

The authors declare that the research was conducted in the absence of any commercial or financial relationships that could be construed as a potential conflict of interest.

## Footnotes

1 https://www.ncbi.nlm.nih.gov/genbank/
