# Supplementary material for: From the sxtA4 Gene to Saxitoxin Production: What Controls the Variability Among Alexandrium minutum and Alexandrium pacificum Strains?
Source: Front Microbiol. 2021 Feb 24;12:613199. doi: 10.3389/fmicb.2021.613199 (PMC7944994; doi:10.3389/fmicb.2021.613199)

Fig. S1: Cell densities for (A) *A. minutum* and (B) *A. pacificum* cultures. All results are averages of triplicate flasks. Errors bars represent standard deviations. The arrows represent the sampling times at the end of the exponential growth phase.

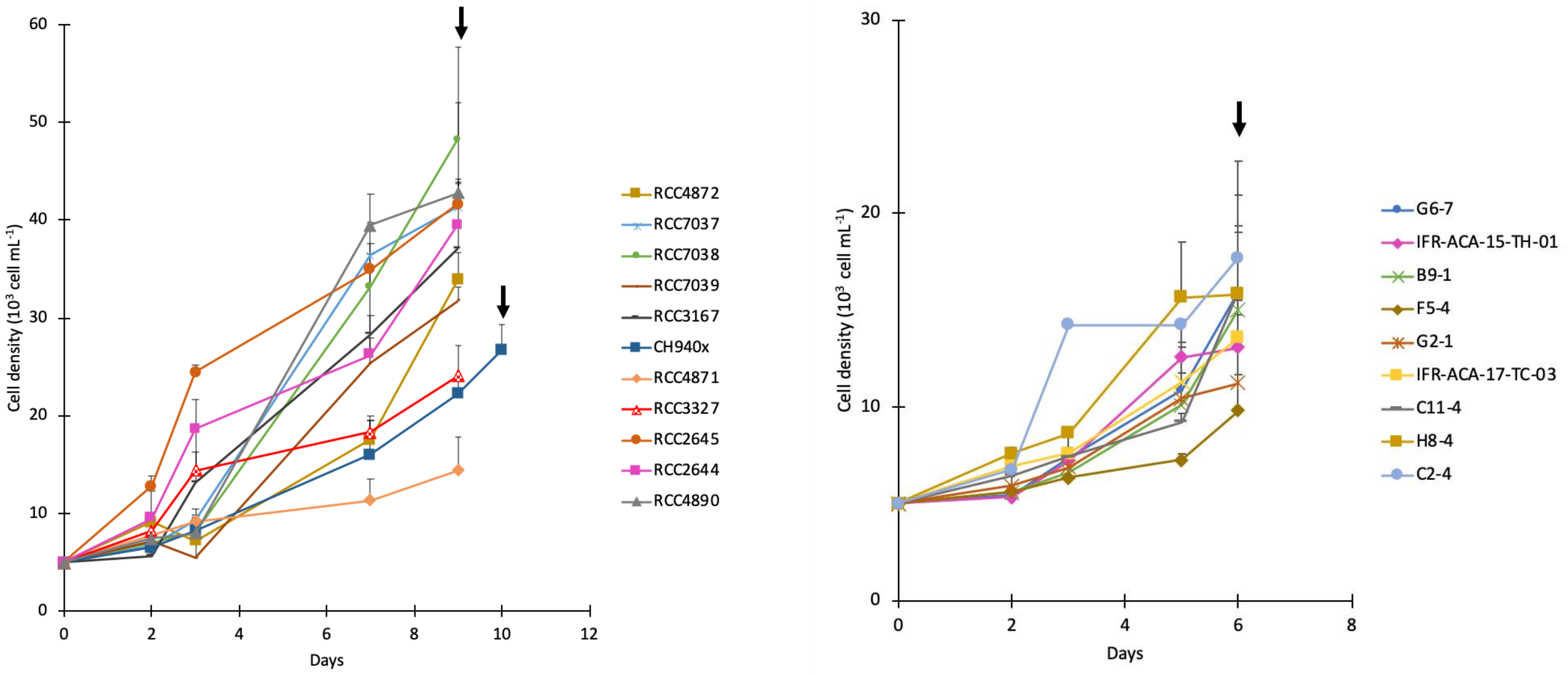

Fig. S2: Scatterplots of the relationship between genome size and *sxtA4* gene copy number **(A)** in *A. minutum* (ρ = -0.43, S = 314, p = 0.19) and **(B)** *A. pacificum* strains (ρ = -0.57, S = 188, p = 0.12).

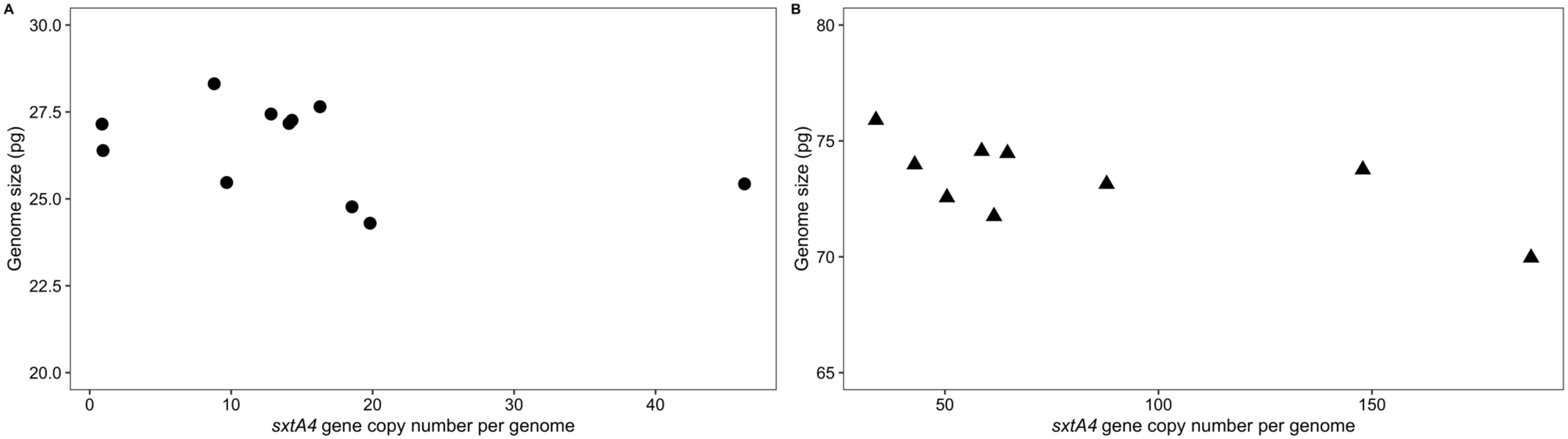

Supplement: Supplementary file 2 [file Data_Sheet_2.PDF]
