# Supplementary material for: From the sxtA4 Gene to Saxitoxin Production: What Controls the Variability Among Alexandrium minutum and Alexandrium pacificum Strains?
Source: Front Microbiol. 2021 Feb 24;12:613199. doi: 10.3389/fmicb.2021.613199 (PMC7944994; doi:10.3389/fmicb.2021.613199)
Supplement: Supplementary file 3 [file Data_Sheet_3.PDF]

Table S1 : BLAST alignment results from LSU partial sequence.

Strains	BLAST results		Accession number
	Description	Percentage identity	
G6-7, IFR-ACA-15, B9-1, C11-4, F5-4, G2-1, C2-4, H8-4, IFR-ACA-17	*A. pacificum* strain CCMP-1493 large subunit ribosomal RNA gene, partial sequence	100%	MK566192.1

Table S2: STX analogues analysed in by HPLC-FLD with limits of detection (LOD) and quantification (LOQ) for each analogue (expressed as µmol L^{-1}).

**STX and analogues**	**LOD (µmol L^{-1})**	**LOQ (µmol L^{-1})**
GTX4	0.030	0,090
GTX1	0.035	0.105
GTX3	0.010	0.030
GTX2	0.015	0.045
dcGTX3	0.003	0.009
dcGTX2	0.012	0.036
GTX5	0.040	0.120
NeoSTX	0.030	0.090
dcSTX	0.018	0.054
STX	0.020	0.060
C1	0.033	0.099
C2	0.018	0.054

Table S3: Primers used in this study.

Gene	Primer name	Forward and reverse primers 5' -> 3'	Product size (pb)	Application	References
sxtA4	sxt007	ATG CTC AAC ATG GGA GTC ATC C	715	Genomic amplification	Stüken et al., 2011
	sxt008	GGG TCC AGT AGA TGT TGA CGA TG			Stüken et al., 2011
	sxt072-MinuPaci	GAC TTG CCC TCC ATA TGT GC	184	Quantitative PCR target	This publication
	sxt073	GCC CGG CGT AGA TGA TGT TG			Stüken et al., 2015

Table S4: Sequence and accession numbers, including several clones of the strains RCC2645 (Genbank Accession No MW546902.1/MW546903.1/MW546904.1), IFR-ACA-15 (Genbank Accession No MW546905.1/MW546906.1) and B9-1 (Genbank Accession No MW546907.1/MW546908.1) used for molecular analyses.

Species	Gene	Accession number
Alexandrium fundyense	*sxtA* domain A4	KJ879208.1
		KJ879196.1
Alexandrium minutum	*sxtA* domain A4	JF343314.1
		JF343312.1
		KM438017.1
		KM438018.1
		JF343348.1
		JF343316.1
		MW546902.1
		MW546903.1
		MW546904.1
Alexandrium pacificum	*sxtA*	JF343329.1
		JF343259.1
	sxtA domain A4	KM100455.1
		KM100453.1
		MW546905.1
		MW546906.1
		MW546907.1
		MW546908.1

Table S5: Protocol of sample preparation for genome size analysis (from Marie et al., 2000 with minor modifications)

For each strain, 20 µL Pluronic F68 (Sigma P1300) was added to 2 mL of exponentially-growing culture and centrifuged for 15 min at 3,000 g. The supernatant was discarded, and the cells were resuspended in 50 µL 0.2 µm-filtered seawater. Then 2 mL 80% ethanol was added and the samples were stored at -20°C. Prior to the analysis, the samples were centrifuged for 15 min at 3,000 g and resuspended in 500 µL of PBS (1X) supplemented with 5 µL of RNAse A (10%) and then incubated at 37°C for 30 min. Human blood cells (HBC, 2C=6.8 Gbp) were used as an internal standard. A blood input volume of 50 µL was injected into 1 mL of nuclei isolation buffer medium (30 mM MgCl2, 20 mM sodium citrate, 120 mM Sorbitol, 55 mM Hepes, 5 mM EDTA, pH 8.0) diluted twice with MilliQ water. Twenty µL of the blood cell suspension was added to each sample before introducing Propidium iodide at 30 µg/mL (final concentration).
